# Potent anti-cancer activity of *Sphaerocoryne affinis* fruit against cervical cancer HeLa cells via inhibition of cell proliferation and induction of apoptosis

**DOI:** 10.1186/s12906-023-04127-0

**Published:** 2023-08-19

**Authors:** Nghia Le-Trung, Tue Minh Duong, Thao Thi Phuong Dang, Kaeko Kamei

**Affiliations:** 1 Department of Functional Chemistry, Kyoto Institute of Technology, Kyoto, 606-8585 Japan; 2 Laboratory of Cancer Research, University of Science, Vietnam National University Ho Chi Minh City, Ho Chi Minh City, Vietnam

**Keywords:** Anti-cancer, Apoptosis, Cervical cancer, DNA damage, HeLa cells, *Sphaerocoryne affinis*

## Abstract

**Background:**

Cervical cancer remains a significant global health issue, highlighting the need for effective therapeutic strategies. Given that *Sphaerocoryne affinis* (SA) has shown potential anti-cancer activity in several cancer types, herein, we investigate the effects of SA fruit (SAF) on human cervical cancer HeLa cells and their underlying mechanisms of action.

**Methods:**

SAF extract cytotoxicity was assessed in various cancer cell lines. The effects of the hexane fraction (SAF-Hex) on HeLa cell viability, cell cycle protein expression, apoptosis, and DNA damage were evaluated using cytotoxicity assays, Western blotting, quantitative PCR, 4′,6-diamidino-2-phenylindole (DAPI) staining, and a terminal deoxynucleotidyl transferase dUTP nick end labeling (TUNEL) assay.

**Results:**

SAF-Hex selectively inhibited HeLa cell viability with an IC50 of 4.20 ± 0.36 µg/mL and a selectivity index of 5.11 ± 0.58. The time-dependent cytotoxicity assay showed decreased cell survival after 48 h of treatment, accompanied by morphological changes and apoptotic bodies in HeLa cells. SAF-Hex also suppressed HeLa cell cycle proteins (Cyclin E, CDK2, and CDK1), reduced *PCNA* transcription, and diminished AKT and mTOR activation, thus inhibiting cell proliferation. The increased γH2AX expression, DNA fragmentation, and caspases-3 and -9 activation indicated SAF-Hex-induced DNA damage and apoptosis. However, the BAX/BCL-2 ratio remained unchanged, and *BAX* and *BCL2* expression was attenuated.

**Conclusion:**

SAF-Hex effectively inhibits HeLa cell proliferation and induces DNA damage in that cervical cancer cell line activating apoptosis through the intrinsic pathway. Interestingly, the BAX/BCL-2 ratio remained unchanged while *BAX* and *BCL2* transcription was attenuated. Hence, further research is required to explore this unexpected finding and facilitate the development of novel therapies targeting cervical cancer HeLa cells.

**Supplementary Information:**

The online version contains supplementary material available at 10.1186/s12906-023-04127-0.

## Background

Cervical cancer ranks as the fourth most common cancer and the leading cause of cancer-related deaths among women [1]. Human papillomavirus (HPV) is the primary factor in cervical cancer development, with smoking, high parity, long-term use of oral contraceptives, and sexually transmitted diseases acting as cofactors. Preventative measures, such as HPV vaccination and screening programs, have led to decreased incidence and death rates. However, low- and middle-income countries continue to experience disproportionately high cervical cancer rates, accounting for ~ 90% of new cases and deaths worldwide in 2020 [2]. Treatment options include surgery, radiation, chemotherapy, and immunotherapy alone or in combination [3].

Currently, the US Food and Drug Administration has approved five drugs for treating cervical cancer, including bleomycin sulfate, topotecan hydrochloride, bevacizumab, pembrolizumab, and tisotumab vedotin-tftv. These drugs target DNA synthesis or specific molecules involved in cancer growth, angiogenesis, immune response, and cell cycle arrest. However, tumor cell drug resistance is a significant cause of therapeutic failure, leading to disease relapse [4]. Moreover, single-drug treatments targeting individual markers or pathways may not effectively cure cervical cancer; therefore, combinatorial therapeutic strategies against specific molecular or cancer markers could prove effective [5].

Plants are abundant sources of substances with anti-cancer potential, with *Sphaerocoryne affinis* (SA) as a prime example. This flowering plant, belonging to the Annonaceae family, is found in tropical and subtropical regions and has been studied for its anti-cancer activity since the 1990s. Various parts of SA, including its leaves, roots, stems, bark, and flowers, have exhibited cytotoxic effects against several cancer cell lines, including A549 (lung carcinoma), MCF-7 (breast carcinoma), HT-29 (colon adenocarcinoma), SK-MEL-5, and Malme-3 M (melanoma) [6–10]. Apart from its anti-cancer properties, the water extract of the SA fruit (SAF) exhibits potent antioxidant capacity in the *Drosophila* model [11], whereas its root demonstrates strong antiplasmodial activity [12]. However, the effects of SA on cervical cancer have not yet been explored.

Given the promising anti-cancer activity of SA against other cancer cell lines, we hypothesize that SAF also exhibits cytotoxic effects against cervical cancer cell lines. Accordingly, this study aims to investigate the potential anti-cancer activity of SAF against HeLa cells and explore its underlying mechanisms of action.

## Methods

### Preparation of plant extract

SAF, known as Chùm Đuông in Vietnamese, was purchased at a country market in Tay Ninh province, Vietnam. Dr. Dang Le Anh Tuan identified from the Laboratory of Botany, Department of Ecology and Evolutionary Biology, Faculty of Biology—Biotechnology, University of Science, VNU-HCMC, Vietnam. A voucher specimen (PHH0004912) is available for reference. Plant collection complied with the IUCN Policy Statement on Research Involving Species at Risk of Extinction and the Convention on the Trade in Endangered Species of Wild Fauna and Flora. A total of 30 kg of fresh fruit was treated with ethanol for seven days, resulting in 2250 g of SAF extract. The obtained extract was partitioned with hexane, ethyl acetate, and water. The crude extract and solvent fractions were then concentrated using a rotary vacuum evaporator and dried via lyophilization.

### Cell culture

All cell lines were purchased from the Japanese Collection of Research Bioresources Cell Bank (JCRB Cell Bank). HT29 cells were cultured in Dulbecco’s Modified Eagle medium (Fujifilm, Tokyo, Japan), Ca Ski cells were cultured in Roswell Park Memorial Institution (Fujifilm, Tokyo, Japan) medium, and HeLa, HEK293, HepG2, and HUH7 cell lines were cultured in Eagle’s Minimum Essential medium (Nacalai, Kyoto, Japan), and supplemented with 10% fetal bovine serum (Biosera, Nuaille, France) and 1% penicillin/streptomycin (Fujifilm, Tokyo, Japan). Cells were maintained in a humidified incubator at 5% CO_2_ and 37 °C.

### Dose-dependent cytotoxicity assay

Cell viability was determined using the CCK8 assay. Briefly, cells (1 × 10^4^ cells/well) were cultured in a 96-well plate overnight. The next day, cells were cultured with extract-containing media for 48 h. Subsequently, the culture broth was replaced with fresh medium containing a 10:1 (v/v) mixture of CCK8 solution (Dojindo, Tabaru, Japan). After 3 h, absorbance was measured at 450 nm using the SH-1200 microplate reader (Corona Electric, Ibaraki, Japan). The relative cell viability was calculated by dividing the absorbance of the samples by that of the control. The half-maximal inhibitory concentrations (IC50) value was determined by interpolating the data from the regression curve between the log of the extract concentration and the cell inhibition index using GraphPad Prism 9 software.

### Time-dependent cytotoxicity assay

Analogous to the dose-dependent cytotoxicity assay, HeLa cells were cultured in a 96-well plate and then exposed to SAF-Hex at the determined IC50 concentration. Cell viability was assessed using the CCK8 assay at 0, 24, 48, and 72 h.

### DAPI staining

HeLa cells (1 × 10^4^ cells/well) were seeded onto a chambered coverglass (Thermo Fisher, Massachusetts, US) and treated with SAF-Hex at the IC50 concentration for 48 h. Cells were washed with phosphate-buffered saline (PBS), fixed with 4% paraformaldehyde (Fujifilm, Tokyo, Japan) for 15 min at room temperature, and permeabilized with 0.05% Triton X-100 (Fujifilm, Tokyo, Japan). Subsequently, cells were incubated with 4′,6-diamidino-2-phenylindole (DAPI) for 15 min and washed with PBS. Finally, fluorescence images were examined using the FLUOVIEW FV10i confocal system (Olympus, Tokyo, Japan).

### TUNEL assay

HeLa cells (1 × 10^4^ cells/well) were seeded onto a chambered coverglass and treated with SAF-Hex at the IC50 concentration for 36 h. The terminal deoxynucleotidyl transferase dUTP nick end labeling (TUNEL) assay was performed using the Apoptosis in situ Detection Kit (Fujifilm, Tokyo, Japan) following the manufacturer’s instructions. Positive cells were detected using the Peroxidase Stain DAB Brown Stain Kit (Nacalai, Kyoto, Japan) and imaged using the IX70 microscope (Olympus, Tokyo, Japan).

### Western blot analysis

Briefly, HeLa cells (2.7 × 10^5^ cells/dish) were seeded in 35-mm dishes. After 24 h, cells were treated with SAF-Hex at concentrations of 0, 5, and 25 µg/mL for 36 h. Whole-cell lysates were prepared using RIPA buffer (Cell Signaling, Massachusetts, US), and protein concentrations were quantified using the Pierce™ BCA Protein Assay Kit (Thermo Fisher, Massachusetts, US). A total of 20 µg of protein was separated using 15% sodium dodecyl sulfate–polyacrylamide gel electrophoresis (SDS-PAGE) gel and transferred to a polyvinylidene difluoride (PVDF) membrane. After blocking with 5% bovine serum albumin for 1 h, the membrane was incubated overnight at 4 °C with the appropriate primary antibodies (Table S1). Membranes were then incubated with secondary antibodies for 1 h at room temperature, washed with PBS and 0.1% Tween-20, and bands were visualized and quantified using Amersham™ ECL Prime Western Blotting Detection Reagent and GelAnalyzer 19.1 (www.gelanalyzer.com).

### Quantitative PCR (qPCR) analysis

Cells were treated with SAF-Hex and cultured to extract mRNA using the miRNeasy kit (Qiagen, Germany). The mRNA quantity was measured using a NanoDrop spectrophotometer (Thermo Fisher). Briefly, 1 µg of total mRNA was reverse transcribed with the Transcriptor Universal cDNA Master kit (Sigma Aldrich, US). Real-time PCR reactions were performed using the FastStart SYBR Green Master kit (Sigma Aldrich), primers, and a 50 ng cDNA template. Primer sequences were a combination of previously established [13, 14] and newly designed sequences (Table S2). qPCR was performed using a Light Cycler 96 instrument (Roche Diagnostics, Germany). The thermal cycling protocol comprised an initial denaturation step at 95 °C for 10 min, followed by 45 cycles of denaturation at 95 °C for 15 s, annealing at specified temperatures for 15 s, and extension at 72 °C for 20 s. Gene expression levels were normalized using the 2^−ΔΔCt^ method, with *GAPDH* as the reference gene.

### Statistical analysis

Statistical analysis was performed using one-way analysis of variance (ANOVA) with GraphPad Prism 9 software, followed by Dunnett’s post hoc test for pairwise comparisons with the control group. The experiments were conducted in three to four independent replicates. Data values are presented as mean ± standard deviation (SD). A *P*-value < 0.05 was considered statistically significant for both the ANOVA and Dunnett’s test.

## Results

### Selective induction of HeLa cell cytotoxicity by SAF-Hex

**Fig. 1:**
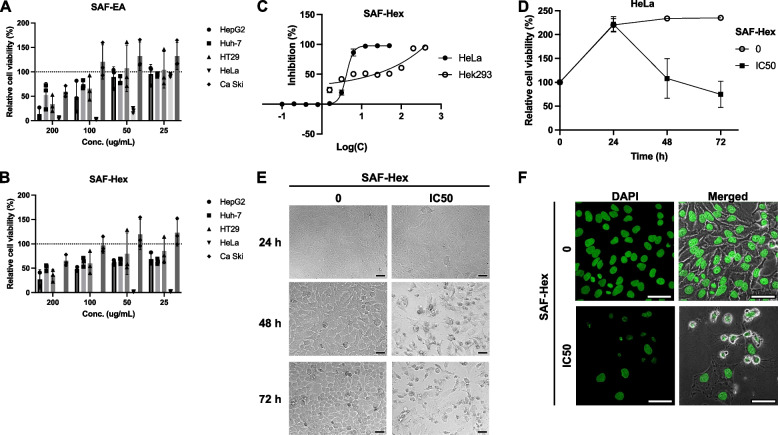
Effects of SAF-Hex on cell cytotoxicity. Relative cell viability of various cell lines following treatment with **A** SAF-EA and **B** SAF-Hex. **C** Dose-dependent cytotoxicity of SAF-Hex, assessed at concentrations ranging from 0 to 100 and 400 µg/mL on HeLa (circle) and HEK293 (square) cells. Relative inhibition of cell survival was plotted against the logarithm of SAF-Hex concentrations (µg/mL). **D** Time-dependent cytotoxicity of SAF-Hex at IC50 (black square) on HeLa cells over 72 h. The control group was cultured without SAF-Hex (white circle). **E** Morphological changes in HeLa cells during the time-dependent cytotoxicity assay at 15 × magnification. **F** Alterations in the nuclei of HeLa cells following treatment with SAF-Hex at the IC50 concentration (4.2 µg/mL), as observed with DAPI staining at 60 × magnification. Scale bars represent 50 μm. IC50, half-maximal inhibitory concentration; SAF-EA, SAF-Hex, ethyl acetate, and hexane fractions of the SAF extract, respectively. Data are represented as mean ± SD; *n* = 3–4

The cytotoxicity of the solvent fractions from the SAF extract was assessed at high doses (25–200 µg/mL) against human cervical cancer (HeLa, Ca Ski), hepatocellular carcinoma (HepG2, Huh-7), and colorectal adenocarcinoma (HT29) cell lines. While the water fraction exhibited no cytotoxic effects on the cell lines (data not shown), the effects of SAF-EA and SAF-Hex are presented in Fig. 1A, B. SAF selectively reduced HeLa cell viability, and SAF-Hex significantly suppressed cancer cell growth. Therefore, further investigations focused on the effects of SAF-Hex on HeLa cells.

A dose-dependent cytotoxicity assay was conducted to analyze the potential of SAF-Hex to inhibit cancer cell growth (Fig. 1C). HeLa (cancerous) and HEK293 (non-cancerous) cells were treated with SAF-Hex at concentrations ranging from 0–100 µg/mL and 400 µg/mL, respectively. After 48 h, the IC50s of SAF-Hex for HeLa and HEK293 cells were 4.20 ± 0.36 and 21.44 ± 2.43 µg/mL, respectively. The selectivity index (SI) was used to assess the selective toxicity of SAF-Hex toward cancer cells compared to normal cells. This was calculated by dividing the IC50 value of HEK293 cells by that of HeLa cells, resulting in an SI value of 5.11 ± 0.58. The time-dependent cytotoxicity assay results (Fig. 1D) indicate cell survival was reduced after 48 h of treatment with SAF-Hex at its IC50 concentration (4.2 µg/mL). During the initial 24 h, HeLa cell growth remained unchanged, with no observable differences in cell viability and morphology between treated and untreated groups (Fig. 1E). However, after 48 h, SAF-Hex-treated cells exhibited abnormal morphology, detaching from the culture plate surface, with shrunken and fragmented nuclei and bubble-shaped bodies (Fig. 1F).

### SAF-Hex inhibits HeLa cell proliferation

**Fig. 2:**
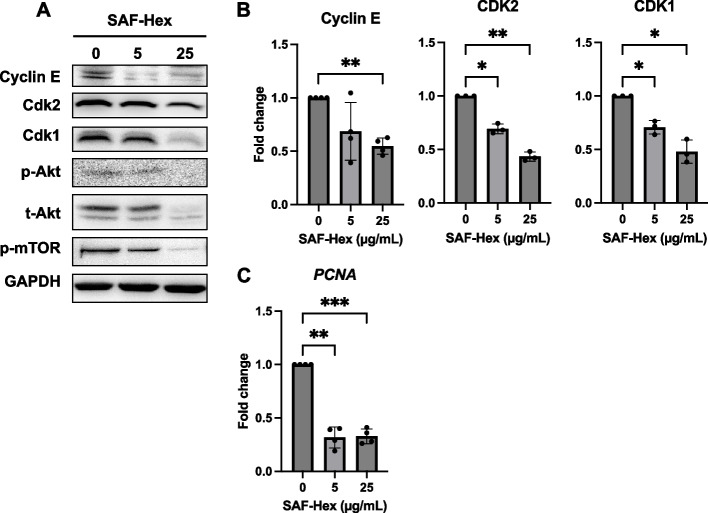
Effects of SAF-Hex on HeLa cell proliferation. HeLa cells were treated with SAF-Hex at concentrations of 0, 5, and 25 µg/mL for 36 h. **A** Western blotting was performed to assess the expression levels of proteins involved in the cell cycle and cell survival. Original images of blots are shown in Fig. S1. **B** Fold changes in expression of Cyclin E, CDK2, and CDK1. **C** Relative transcription levels of *PCNA*, as determined using quantitative PCR. SAF-Hex, hexane fraction of the SAF extract. Data are represented as mean ± SD and analyzed by one-way ANOVA; *P < 0.05, **P < 0.01, ***P < 0.001; *n* = 3–4

Considering that treatment with SAF inhibited cell proliferation, we examined the expression of cyclin E, CDK2, and CDK1, which are essential for cell cycle phase transition [15]. The expression of these proteins was significantly reduced in the SAF-Hex-treated group compared to the control group (Fig. 2A, B). Protein kinase B (AKT) and its downstream target, the mammalian target of rapamycin (mTOR), which promotes cell survival and block apoptosis [16], exhibited decreased activation. Moreover, the proliferating cell nuclear antigen (*PCNA*) gene transcription, which is critical for cells to replicate their genetic material and enter the next cell cycle phase, was also reduced (Fig. 2C).

### SAF-Hex induces DNA damage in HeLa cells

**Fig. 3:**
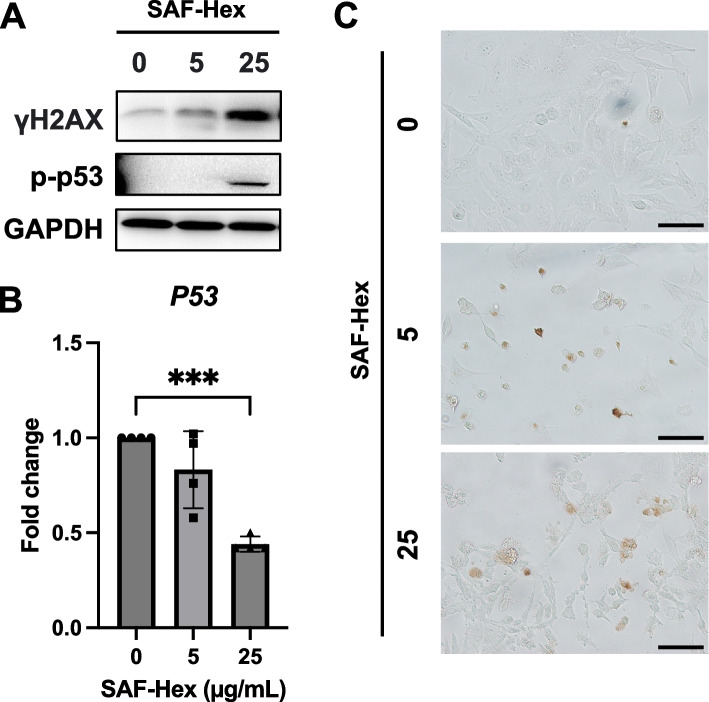
SAF-Hex induces DNA damage in HeLa cells. DNA damage was assessed in HeLa cells treated with SAF-Hex at concentrations of 0, 5, and 25 µg/mL for 36 h using **A** Western blotting for γH2AX and **B** qPCR for *P53* mRNA levels. Original images of Western blots are shown in Fig. S2. **C** TUNEL assay. DNA-fragmented positive cells appear brown. Images were captured at a magnification of 20 × , with scale bars indicating 50 μm. qPCR, quantitative PCR; SAF-Hex, hexane fraction of the SAF extract. Data are represented as mean ± SD and analyzed using one-way ANOVA; *n* = 3–4; ***P < 0.001

The histone variant γH2AX, a marker of DNA damage, is phosphorylated at serine 139 following DNA double-strand breakage [17]. Western blotting was employed to estimate the expression of γH2AX in HeLa cells treated with SAF-Hex at concentrations of 0, 5, and 25 µg/mL (Fig. 3A). The γH2AX levels significantly increased after 36 h of SAF-Hex treatment, and P53 was consequently activated in response to DNA damage. However, the transcription of *P53* mRNA was reduced in the SAF-Hex-treated group compared to the control group (Fig. 3B). TUNEL assay results further revealed increased DNA fragmentation in cells cultured with the extract (Fig. 3C).

### SAF-Hex activates apoptosis on HeLa cells

**Fig. 4:**
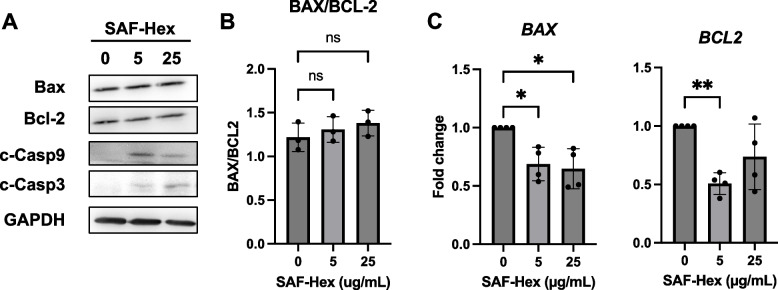
SAF-Hex induces apoptotic cell death in HeLa cells. HeLa cells were treated with SAF-Hex at concentrations of 0, 5, and 25 µg/mL for 36 h. **A** Western blotting was used to measure apoptosis-related protein expression. Original images of blots are shown in Fig. S3. **B** Relative ratio of BAX and BCL-2, as determined using Western blot results. **C** qPCR was utilized to determine the relative transcription levels of *BAX* and *BCL2*. qPCR, quantitative PCR; SAF-Hex, hexane fraction of the SAF extract. Data are represented as mean ± SD and analyzed using one-way ANOVA; *P < 0.05, **P < 0.01; ns, not significant; *n* = 3–4

DNA fragmentation is indicative of apoptosis [18], and caspase-3 has been identified as a crucial effector enzyme in apoptosis [19]. Procaspase-3 is activated through cleavage of its proenzyme form, after which it can catalyze other proteins, leading to cell death [20, 21]. Hence, Western blotting was utilized to examine caspase-3 cleavage activation (Fig. 4A). SAF-Hex treatment increased the expression of cleaved-caspase-3 (17 kDa). Moreover, activation of caspase-9, but not caspase-8, was observed (Fig. S4), both of which are involved in activating caspase-3. However, the ratio of pro-apoptotic protein BAX and anti-apoptotic protein BCL-2 remained unchanged in the treated group compared to the control group (Fig. 4B), while the transcription levels of *BAX* and *BCL2* mRNA decreased (Fig. 4C).

## Discussion

The incidence of cancer is increasing globally, with tropical countries’ biodiversity offering valuable resources for potential cancer treatment candidates. In previous studies, SA, primarily distributed in Southeast Asia and sub-equatorial nations, has demonstrated anti-cancer activity against various cancer cell lines [6–8, 10, 22]. Our present study assessed the anti-cancer properties of SAF. A preliminary screening of five cervical cancer (HeLa, Ca Ski), liver cancer (HepG2, HUH7), and colorectal cancer (HT29) cell lines revealed the selective inhibitory effect of SAF-Hex on HeLa cells. The IC50 value was 4.20 ± 0.36 µg/mL, indicating robust cytotoxicity [23].

The SI measures a treatment’s effectiveness in targeting cancer cells while sparing normal cells. It is defined as the IC50 ratio between normal and cancer cells, with a higher SI value indicating more effective and safer therapy for healthy cells. A promising sample should have an SI value ≥ 3, and samples with SI values ≥ 10 warrant further investigation [24]. In our study, the SI value was 5.11 ± 0.58, as determined using the IC50 of SAF-Hex on HeLa and human embryonic kidney H293 cells. These findings suggest that SAF has high potential for future anti-cancer research. The time-dependent cytotoxicity assay revealed no significant differences in cell viability between both cell groups during the first 24 h, possibly indicating a delayed effect of the anti-cancer treatment on cells. However, HeLa cells detached from the culture surface and exhibited abnormal morphologies at 48 and 72 h post-treatment. SAF-Hex-treated cells displayed shrunken nuclei with uneven distribution and the presence of spheres, which could be apoptotic bodies, implying apoptotic cell death [25].

Since 1990, in-depth studies exploring the effects of SA extracts on cancer cells have been lacking. We hypothesized the following primary mechanism of action of the extract on the HeLa cell line. First, the delayed action of the SAF extract might be related to inhibited cell proliferation. Cyclin E binds to protein kinase CDK2, regulating cell cycle entry into the DNA synthesis phase [26], while CDK1 functions during the G2 phase. SAF-Hex suppressed the expression of these proteins after 36 h, effectively blocking the cell cycle. Additionally, PCNA is a cofactor of DNA polymerase δ, an enzyme complex in eukaryotes involved in DNA replication and repair [27]. Our qPCR analysis showed reduced *PCNA* expression in cells treated with SAF-Hex. The AKT protein, a crucial cellular signaling molecule, plays a significant role in regulating various biological processes, including cell survival, growth, and metabolism [28]. More specifically, AKT inhibits caspase-9 activation and anti-apoptotic BCL-2 while promoting cell proliferation by phosphorylating its downstream target, mTOR. SAF-Hex diminished the activation of p-AKT and p-mTOR in HeLa cells. Consequently, SAF-Hex’s inhibition of cell proliferation can be attributed to its suppression of vital cell cycle proteins, downregulation of *PCNA* expression, and reduced AKT and mTOR activation, ultimately impeding HeLa cell growth.

The histone variant γH2AX serves as an indicator of DNA damage [29]. In our study, γH2Ax expression increased in cells cultured with SAF-Hex, indirectly supporting DNA damage induction by the extract. Additionally, γH2Ax is upregulated during the initiation of DNA fragmentation in apoptosis [30]. The TUNEL assay results showed that SAF-Hex induced DNA fragmentation, an apoptotic marker [18], when culturing HeLa cells with SAF-Hex. Meanwhile, P53 rapidly responds to such damage. Following double-stranded DNA breakage, P53 undergoes activation via post-translational modifications, including phosphorylation, acetylation, methylation, ubiquitination, or sumoylation [31, 32]. These modifications facilitate P53 binding to specific DNA sequences, promoting respective target gene transcription and enhancing the P53 protein abundance by 3–tenfold [33]. Despite increased phosphorylated P53 levels following treatment of HeLa cells with SAF-Hex, *P53* transcript expression decreased by nearly 50% in cells treated with 25 µg/mL SAF-Hex for 36 h. This decline could be attributed to a negative feedback loop [33]. Ljungman et al. observed that mRNA synthesis is blocked following DNA damage caused by ultraviolet exposure [34], elucidating the reduced P53 transcription level at 36 h.

Apoptosis involves various distinct caspases, including caspase-3 [21]. For caspase-3 to become activated, procaspase-3 (32 kDa) must undergo cleavage into a large subunit (17–19 kDa) and small subunit (12 kDa) [35]. HeLa cells treated with SAF-Hex extract exhibited increased expression of cleaved-caspase-3. Moreover, cleaved-caspase-9 is an upstream activator of cleaved-caspase-3 in the intrinsic apoptosis pathway, while cleaved-caspase-8 is involved in the extrinsic pathway. We observed only an increase in activated caspase-9, suggesting that SAF-Hex induces HeLa cell death via the intrinsic apoptosis pathway. Caspase-9 activation is closely related to the loss of mitochondrial outer membrane potential (MOMP), leading to cytochrome c release, forming the apoptosome complex, and subsequently activating caspase-9 [36]. MOMP is triggered by the upregulation and translocation of BAX from the cytosol to the MOM, concomitant with BCL-2 inhibition, resulting in an increased BAX/BCL-2 ratio. In our study, this ratio remained unchanged after 36 h of SAF-Hex treatment. This unusual occurrence has also been reported when exposing HeLa cells to the Newcastle disease virus, leading to apoptosis while maintaining a constant BAX/BCL-2 ratio [37]. Meanwhile, we observed attenuated *BAX* and *BCL2* gene transcription levels in the SAF-Hex-treated group compared to the control group. Arıcan et al. [38] observed a similar phenomenon when treating HeLa cells with *Astragalus* L. root extract for 72 h. Although the precise cause of these results is unclear, we postulate that this decrease could result from a negative feedback loop or high cytotoxicity.

## Conclusions

In conclusion, our study demonstrated the potent anti-cancer properties of SAF on HeLa cells, with a selective inhibitory effect and delayed action mechanism. The results suggest that SAF-Hex inhibits cell proliferation by suppressing vital cell cycle proteins, downregulating *PCNA* transcription, and reducing AKT and mTOR activation. Furthermore, SAF-Hex induces DNA damage and apoptosis in HeLa cells through the intrinsic pathway, as evidenced by the increased expression of γH2AX, activation of P53, caspase-3, and caspase-9, and DNA fragmentation. Although the BAX/BCL-2 ratio remained constant, these findings highlight the potential of SAF as a promising candidate for further study in the development of cancer therapies, particularly targeting cervical cancer HeLa cells.

### Supplementary Information

**Additional file 1: Fig. S1.** Original images of blots shown in Fig. 2A. **Fig. S2.** Original images of blots shown in Fig. 3A. **Fig. S3.** Original images of blots shown in Fig. 4A. **Fig. S4.** Western blotting of caspase-8.

**Additional file 2: Table S1.** Antibodies for Western Blot Assay. **Table S2.** Primers for qPCR.

## Data Availability

The datasets generated and/or analyzed during the current study are available from the corresponding author on reasonable request.

## Abbreviations

AKT: Protein kinase B
CCK8: Cell counting kit-8
CDKs: Cyclin Dependent Kinases
DAPI: 4′,6-Diamidino-2-phenylindole
H2AX: H2A histone family member X
HPV: Human papillomavirus
IC50: Half-maximal inhibitory concentrations
MOMP: Mitochondrial outer membrane potential
mTOR: Mammalian target of rapamycin
PBS: Phosphate-buffered saline
PCNA: Proliferating cell nuclear antigen
PVDF: Polyvinylidene difluoride
qPCR: Quantitative polymerase chain reaction
SA: *Sphaerocoryne affinis*
SAF: SA fruit
SD: Standard deviation
SI: Selectivity index
TUNEL: Terminal deoxynucleotidyl transferase dUTP nick end labeling
