# Supplementary material for: Potent anti-cancer activity of Sphaerocoryne affinis fruit against cervical cancer HeLa cells via inhibition of cell proliferation and induction of apoptosis
Source: BMC Complement Med Ther. 2023 Aug 19;23:290. doi: 10.1186/s12906-023-04127-0 (PMC10439542; doi:10.1186/s12906-023-04127-0)
Supplement: Supplementary file 1 — Additional file 1: Fig. S1. Original images of blots shown in Fig. 2A. Fig. S2. Original images of blots shown in Fig. 3A. Fig. S3. Original images of blots shown in Fig. 4A. Fig. S4. Western blotting of caspase-8. [file 12906_2023_4127_MOESM1_ESM.pdf]

Fig. S1: Original images of blots shown in Fig. 2A

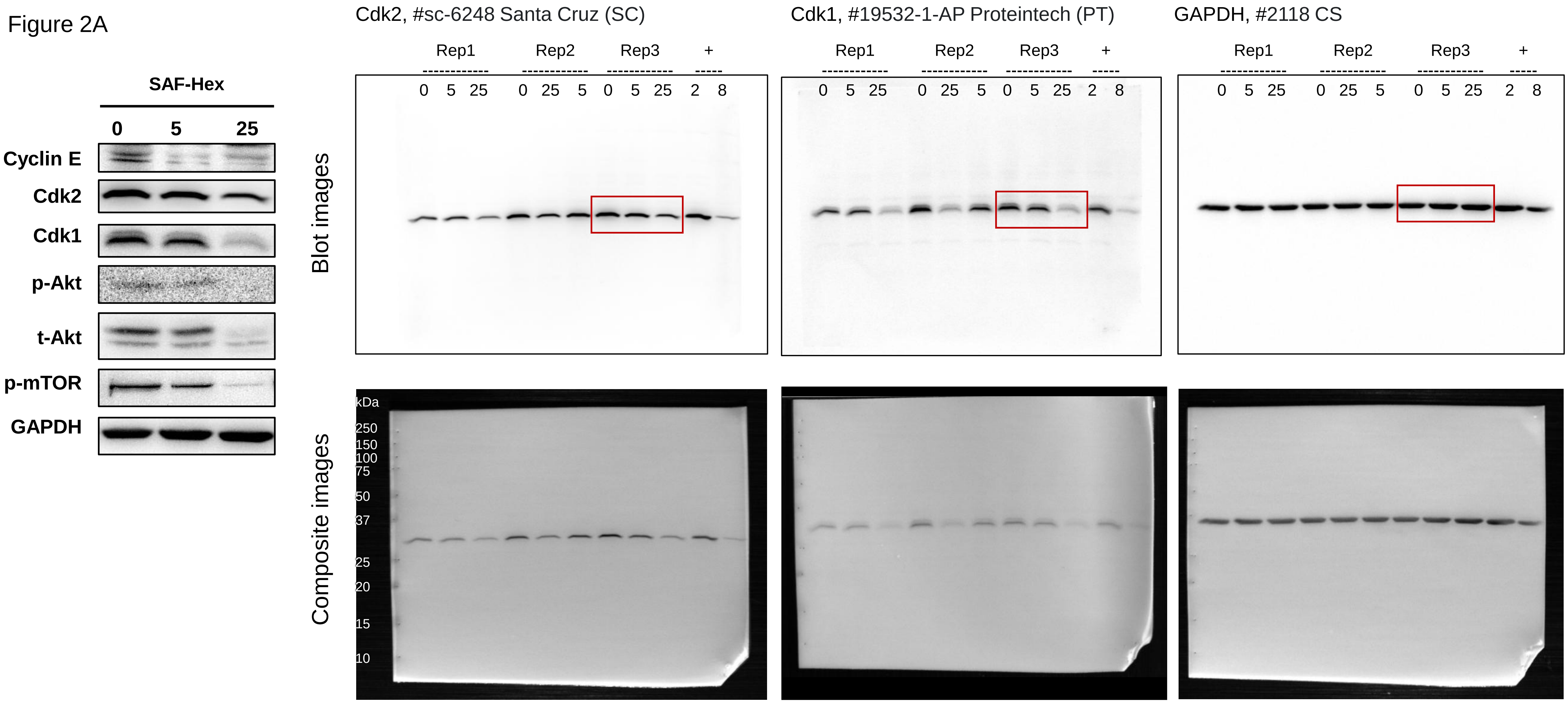

+, Staurosporine 1 µM; 2, 2 h; 8, 8 h

Boxed bands are shown in Fig. 2A as representives.

Fig. S1 (continued): Original images of blots shown in Fig. 2A

Figure 2A

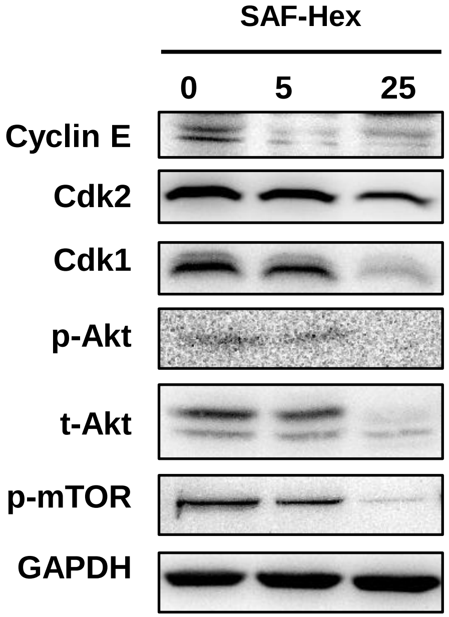

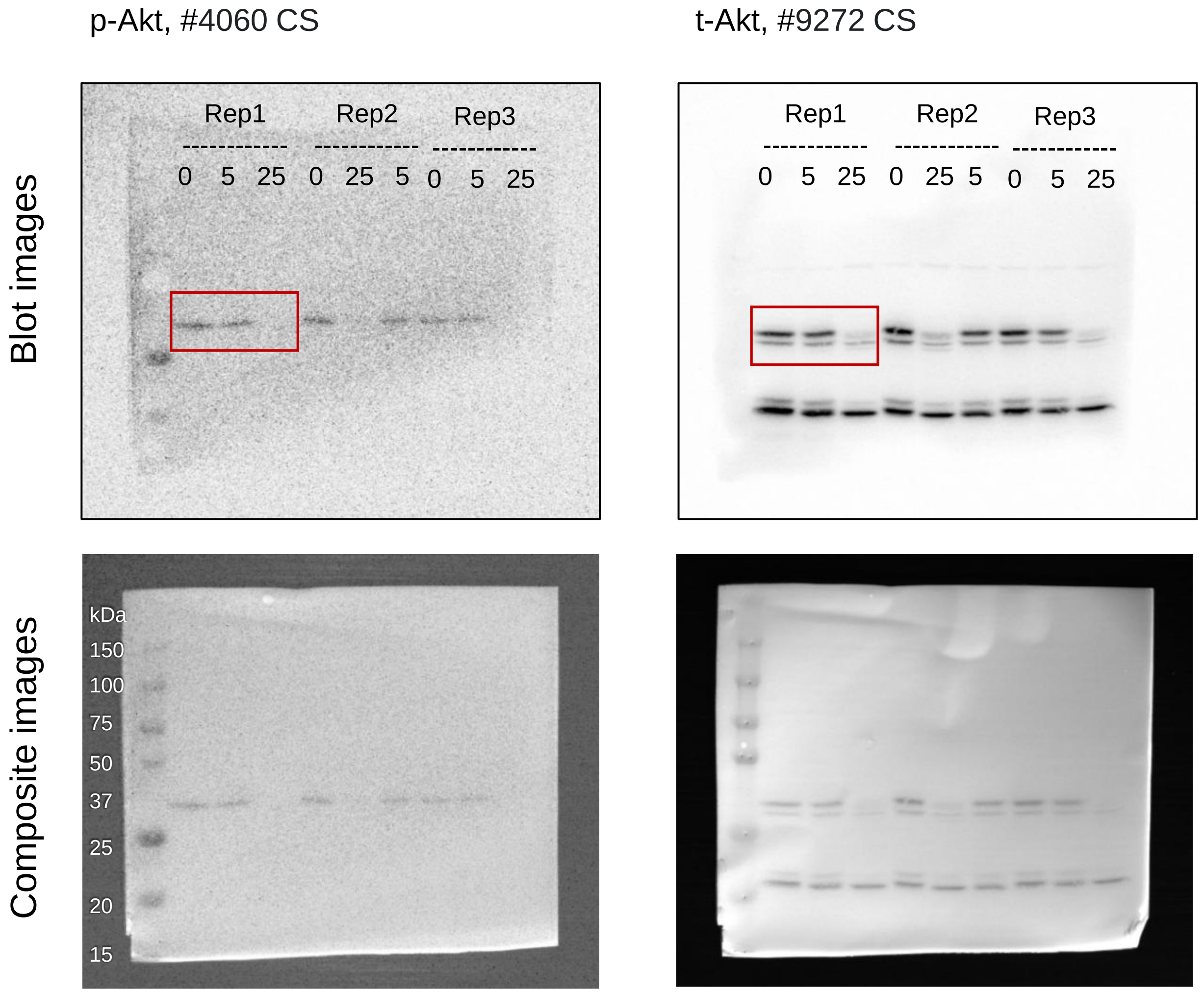

Boxed bands are shown in Fig. 2A as representives.

Fig. S1 (continued): Original images of blots shown in Fig. 2A

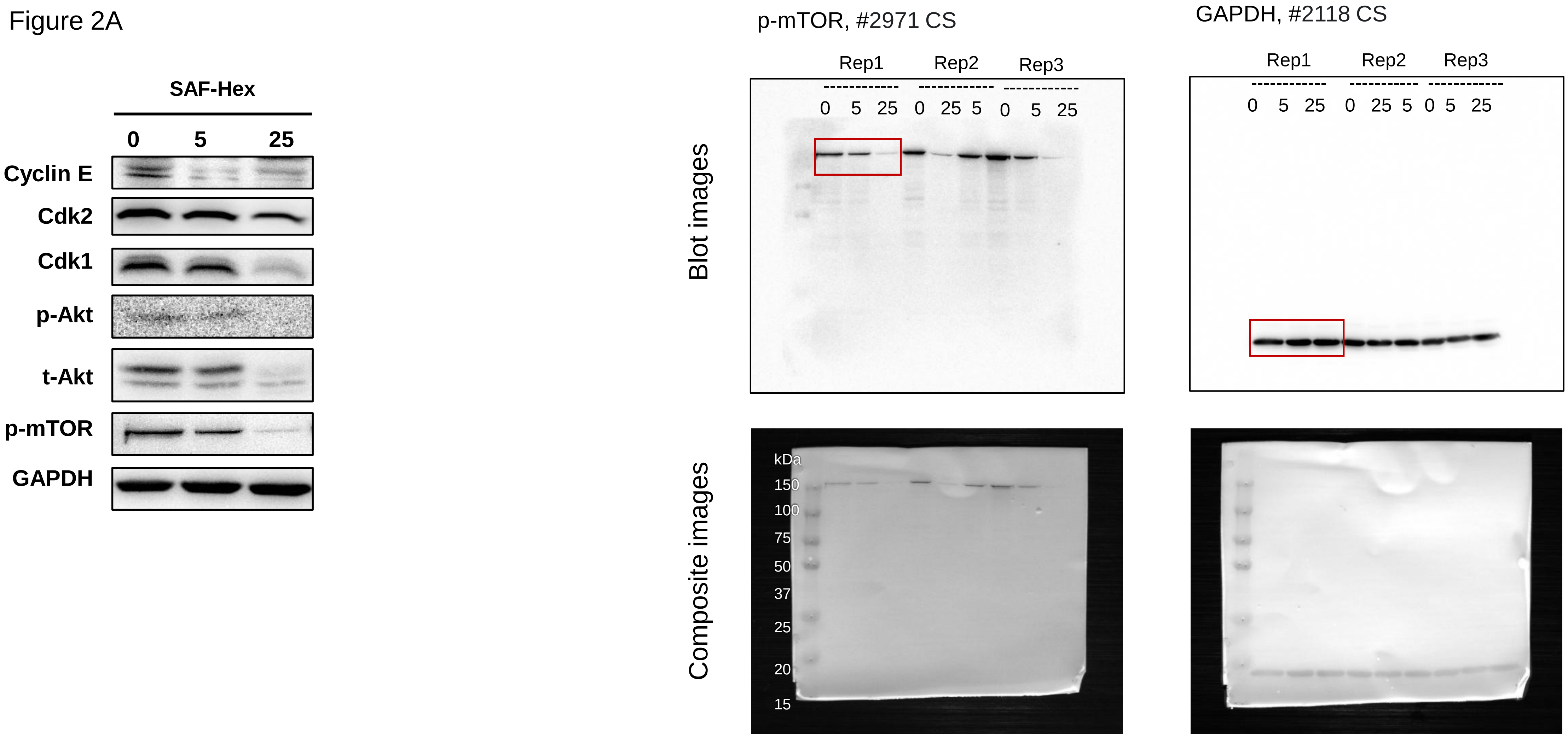

Boxed bands are shown in Fig. 2A as representives.

Fig. S1 (continued): Original images of blots shown in Fig. 2A

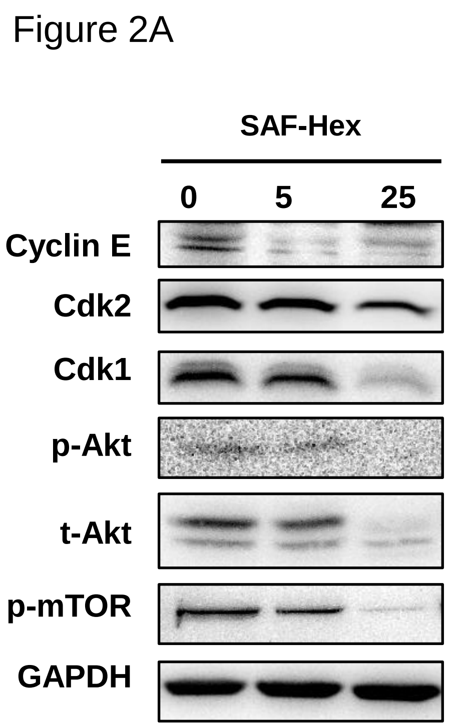

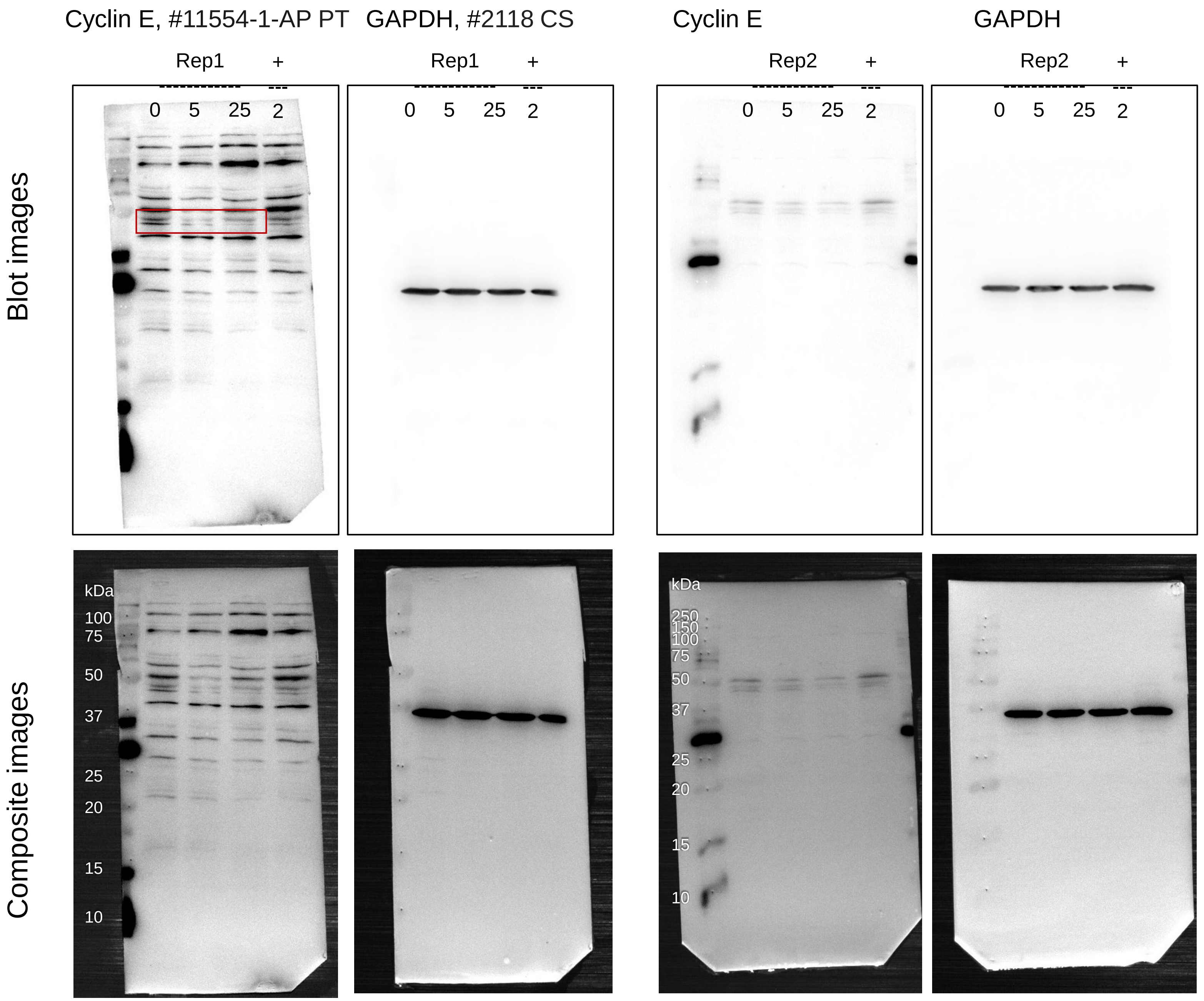

+, Staurosporine 1 μM; 2, 2 h

Boxed bands are shown in Fig. 2A as representives.

Fig. S1 (continued): Original images of blots shown in Fig. 2A

Figure 2A

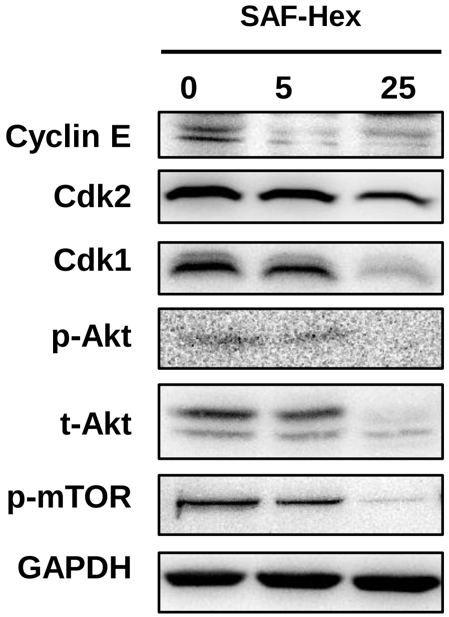

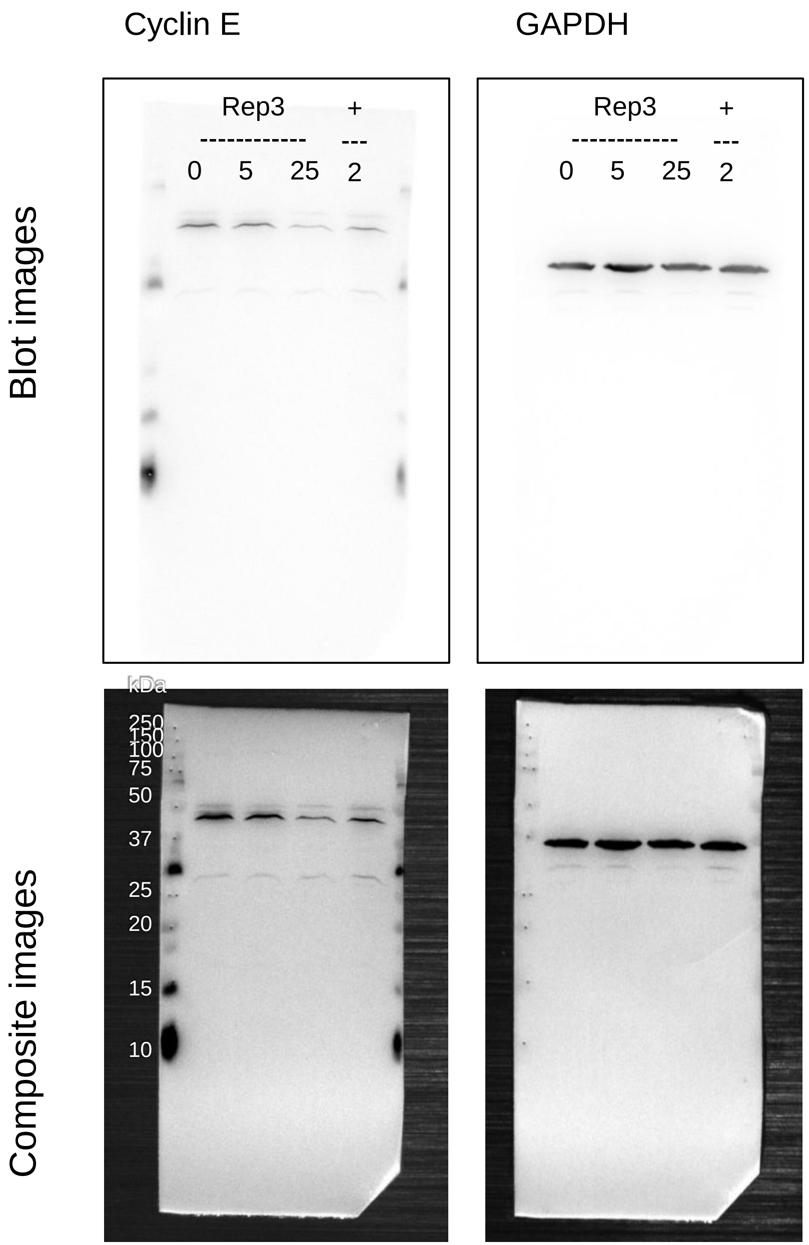

+, *Staurosporine 1 μM; 2, 2 h*

Boxed bands are shown in Fig. 2A as representives.

Fig. S2: Original images of blots shown in Fig. 3A

Figure 3A

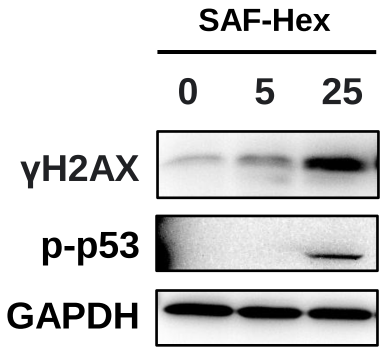

p-p53, #645501 Cell Signaling (CS)

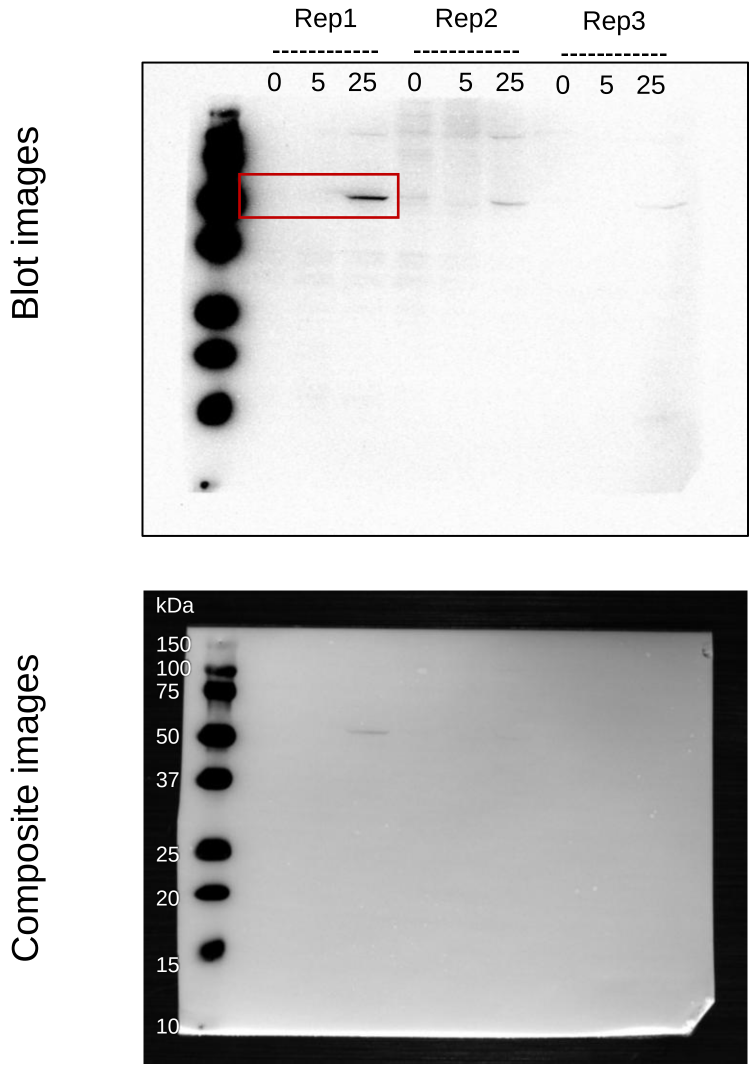

GAPDH, #2118 CS

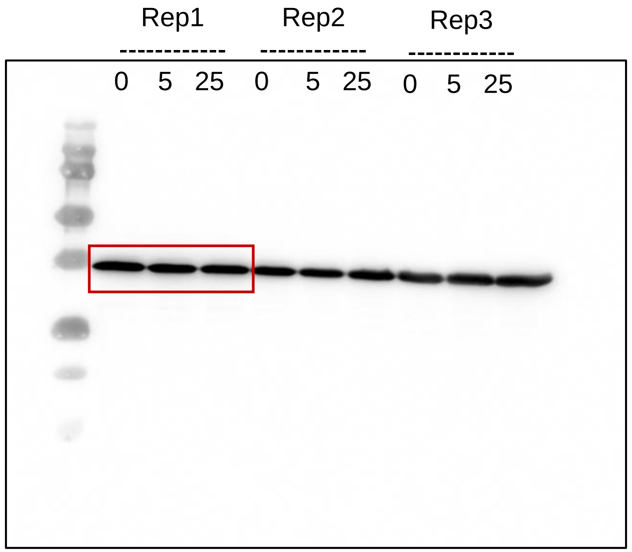

Unavailable

Boxed bands are shown in Fig. 3A as representives.

Fig. S2 (continued): Original images of blots shown in Fig. 3A

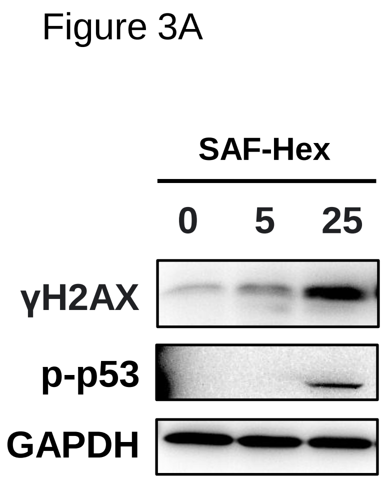

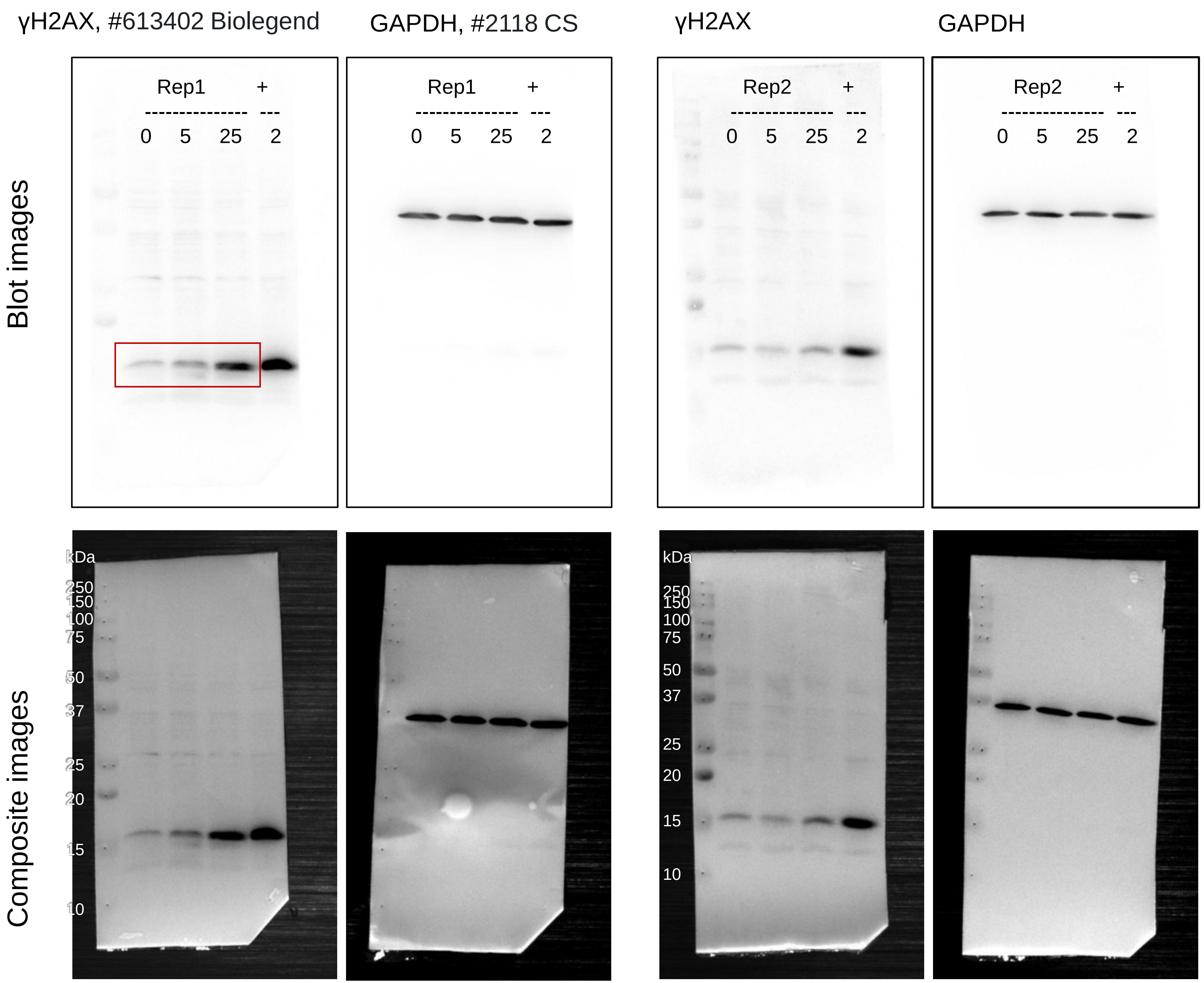

+, Staurosporine 1 μM; 2, 2 h

Boxed bands are shown in Fig. 3A as representives.

Fig. S2 (continued): Original images of blots shown in Fig. 3A

Figure 3A

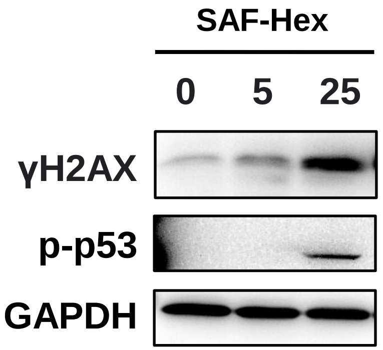

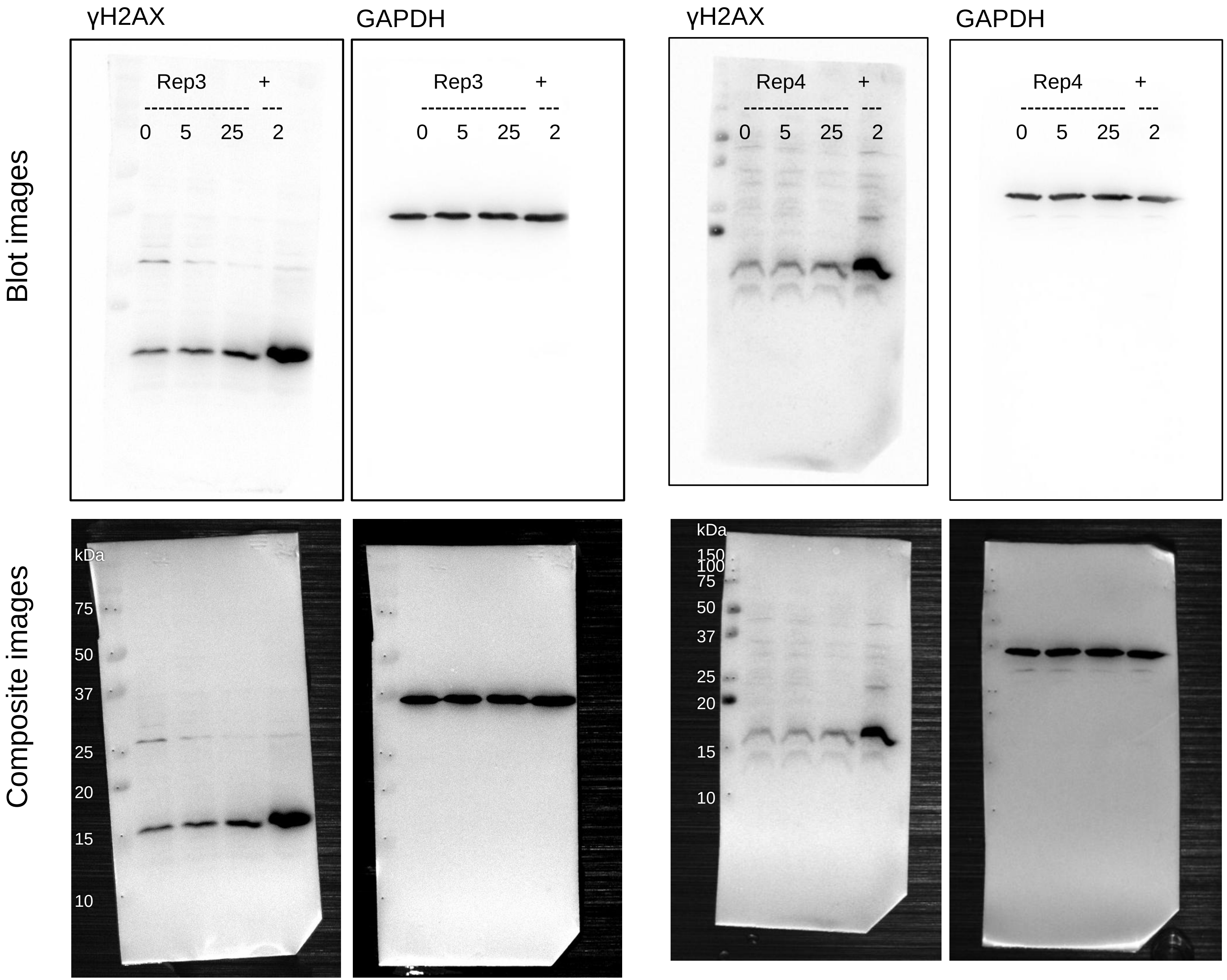

+, *Staurosporine 1 μM; 2, 2 h*

Boxed bands are shown in Fig. 3A as representives.

Fig. S3: Original images of blots shown in Fig. 4A

Figure 4A

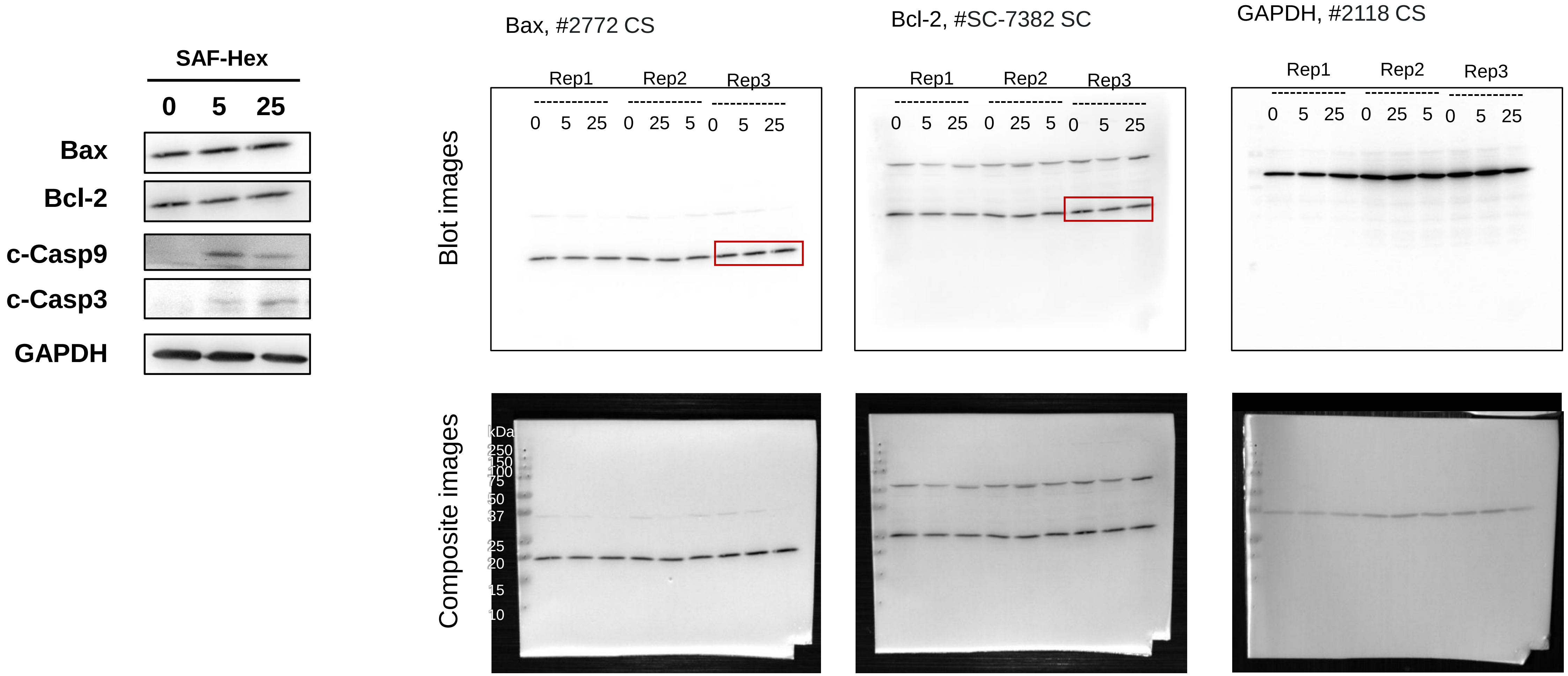

Boxed bands are shown in Fig. 4A as representives.

Fig. S3 (continued): Original images of blots shown in Fig. 4A

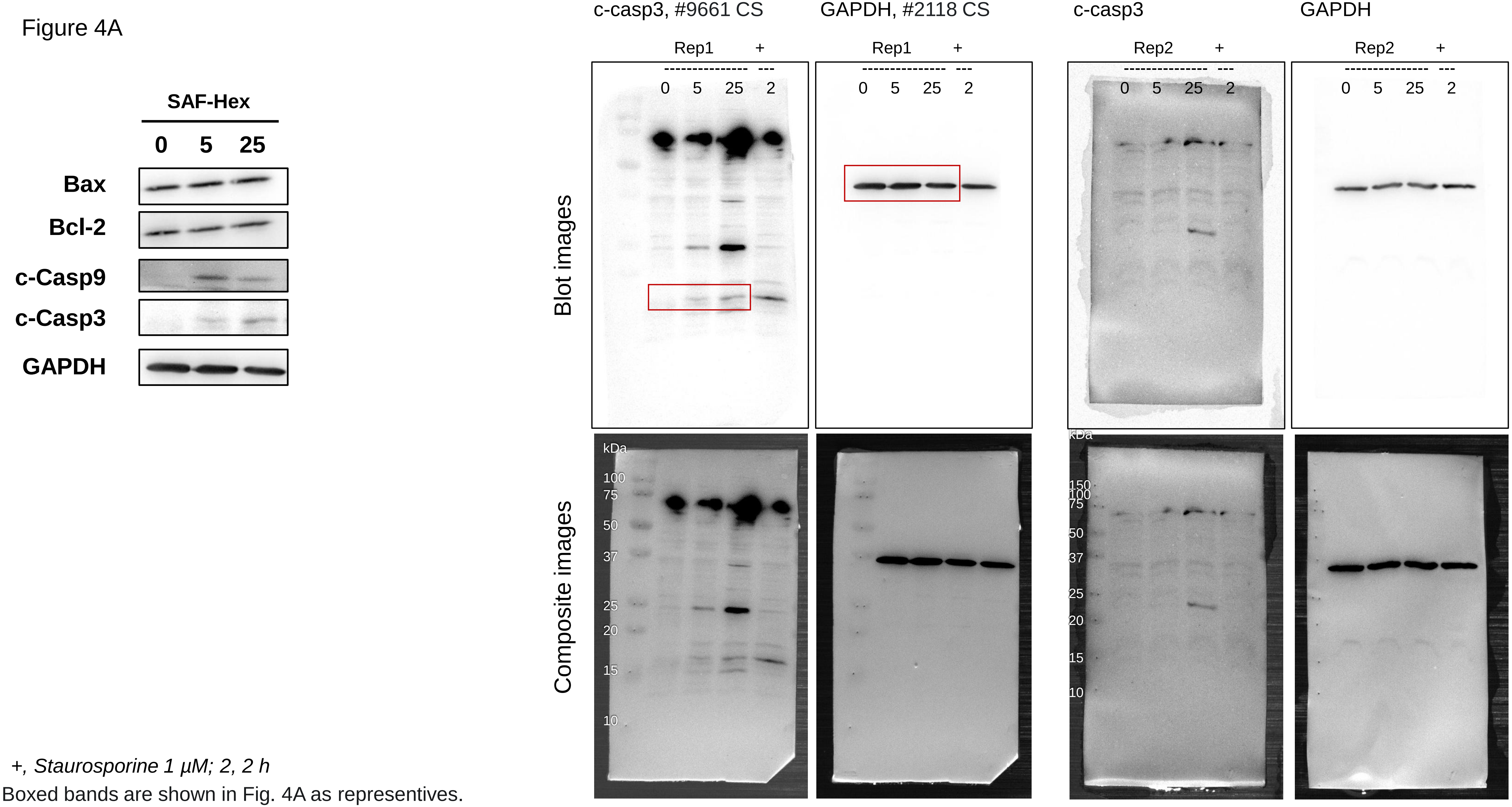

+, *Staurosporine 1 μM; 2, 2 h*

Boxed bands are shown in Fig. 4A as representives.

Fig. S3 (continued): Original images of blots shown in Fig. 4A

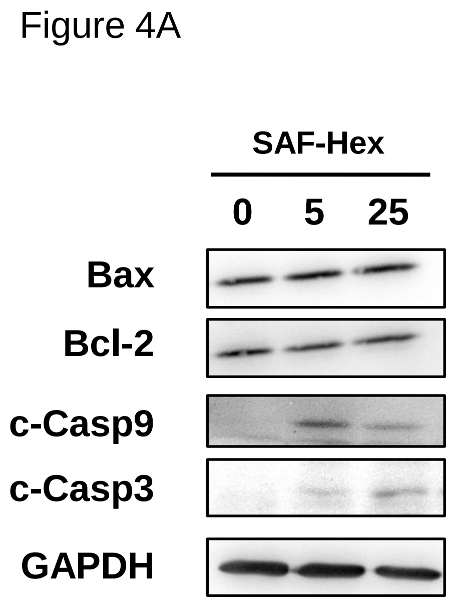

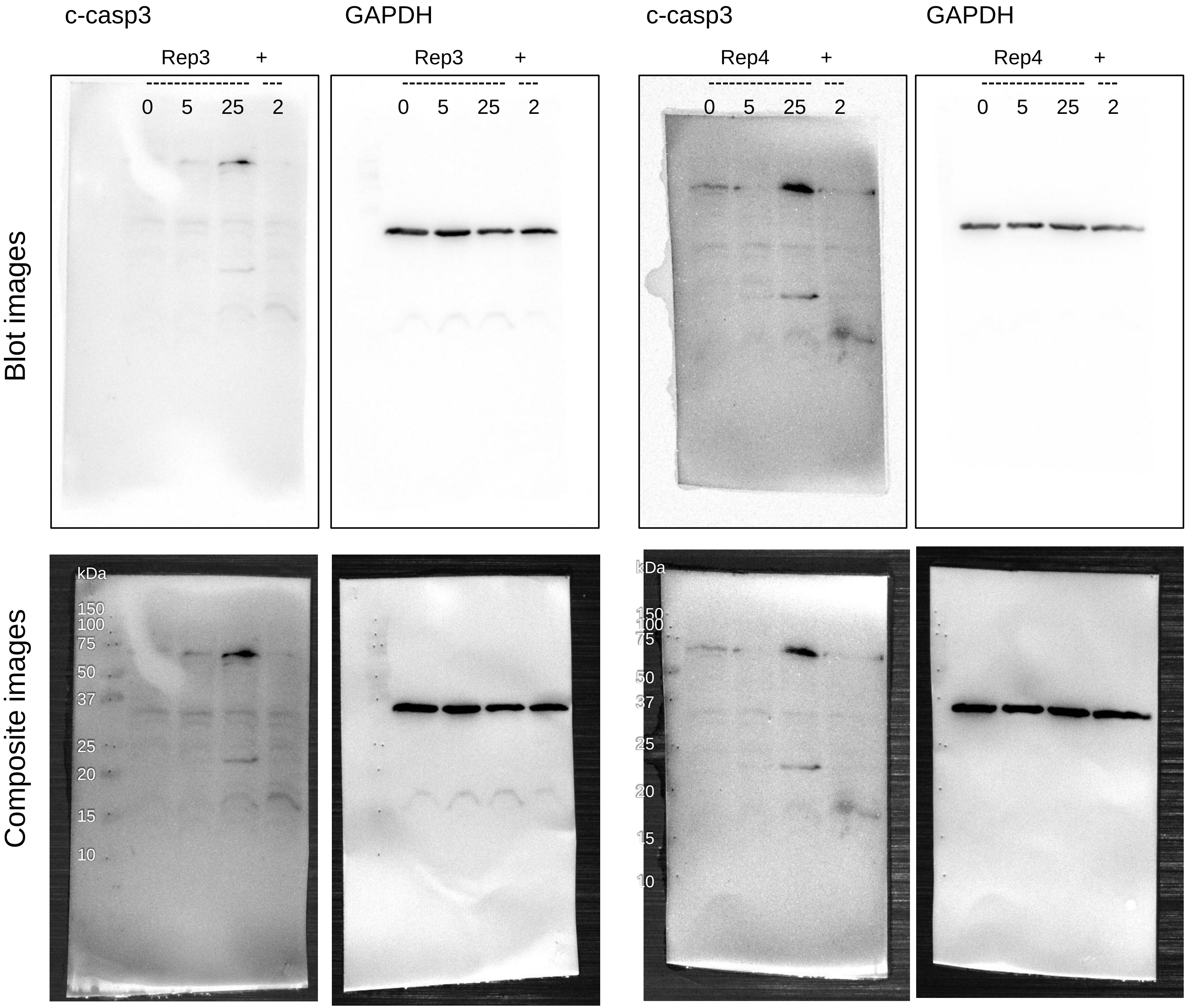

+, *Staurosporine 1 μM; 2, 2 h*

Boxed bands are shown in Fig. 4A as representives.

Fig. S3 (continued): Original images of blots shown in Fig. 4A

Figure 4A

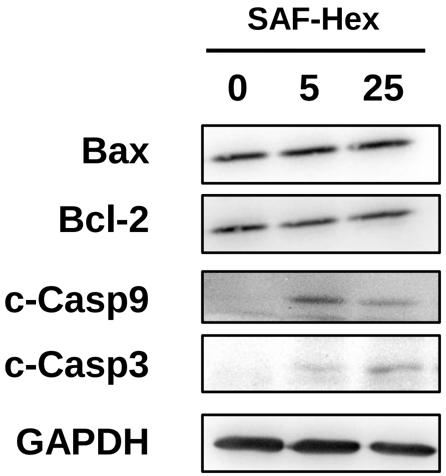

c-caspase-9, #7237 CS

GAPDH, #2118 CS

Blot images

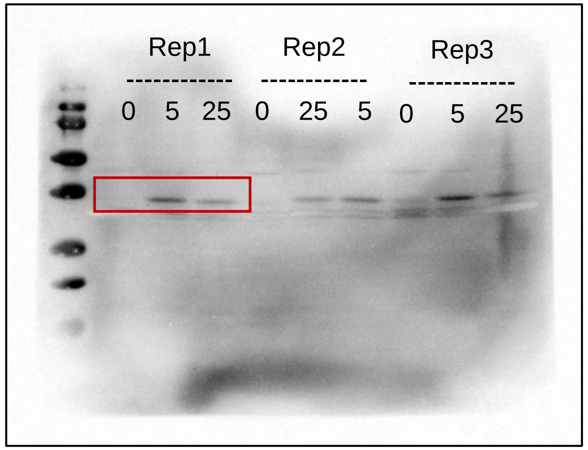

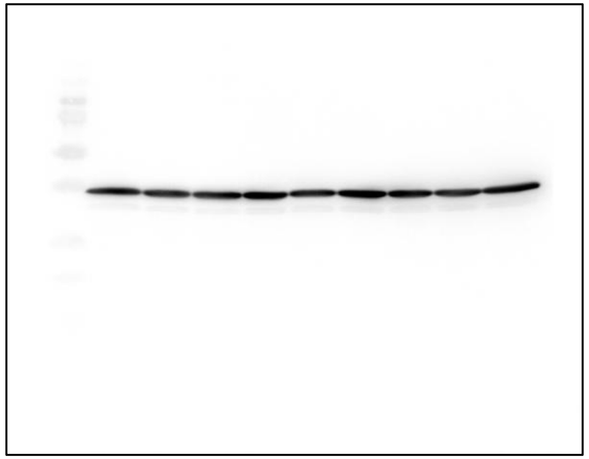

Composite images

Unavailable

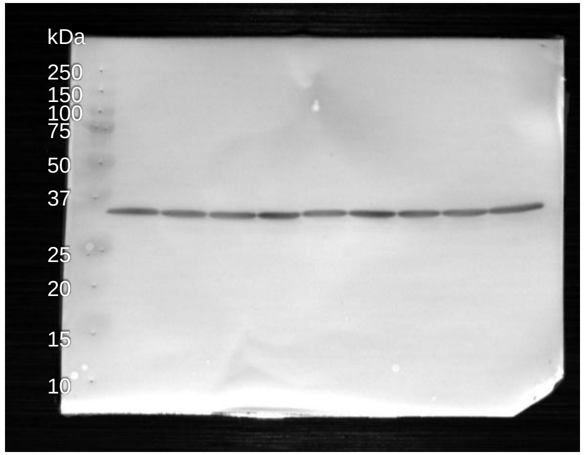

Boxed bands are shown in Fig. 4A as representives.

Fig. S4: Western blotting of caspase-8

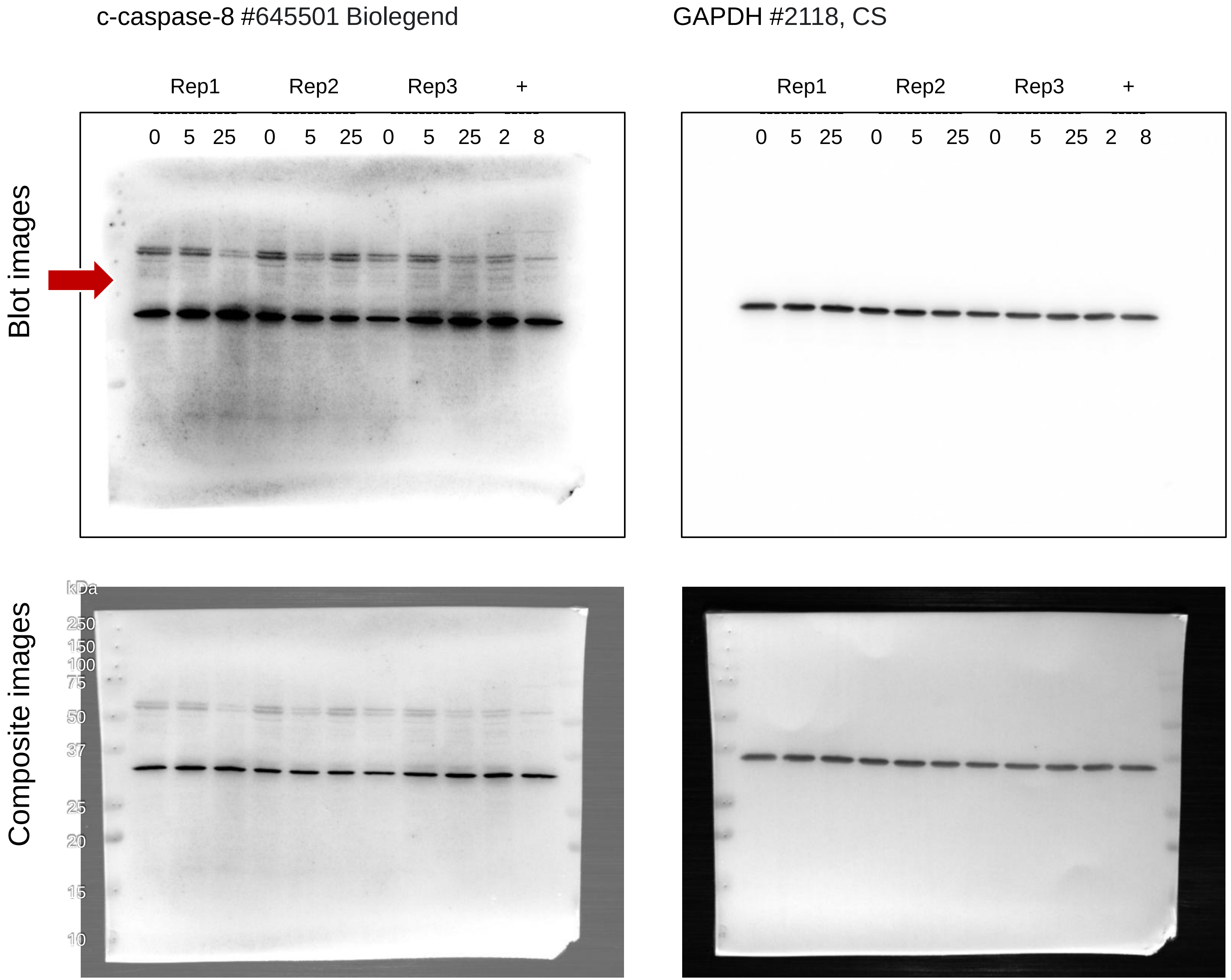

The red arrow indicates caspase-8.
