# Supplementary material for: Potent anti-cancer activity of Sphaerocoryne affinis fruit against cervical cancer HeLa cells via inhibition of cell proliferation and induction of apoptosis
Source: BMC Complement Med Ther. 2023 Aug 19;23:290. doi: 10.1186/s12906-023-04127-0 (PMC10439542; doi:10.1186/s12906-023-04127-0)
Supplement: Supplementary file 2 — Additional file 2: Table S1. Antibodies for Western Blot Assay. Table S2. Primers for qPCR. [file 12906_2023_4127_MOESM2_ESM.pdf]

Table S1. Antibodies for Western Blot Assay

**Antibody**	**Dilution rate**	**Code**	**Manufacturer**
Anti-BAX	1:1000	2772	Cell Signaling
Anti-BCL-2	1:1000	SC-7382	Santa Cruz Biotech
Anti-c-Casp3	1:1000	9661	Cell Signaling
Anti-c-Casp9	1:1000	7237	Cell Signaling
Anti-Cyclin E	1:4000	11554-1-AP	Proteintech
Anti- γH2AX	1:4000	613402	Biolegend
Anti-CDK2	1:1000	SC-6248	Santa Cruz Biotech
Anti-CDK1	1:1000	19532-1-AP	Proteintech
Anti-p-AKT	1:1000	4060	Cell Signaling
Anti-t-AKT	1:1000	9272	Cell Signaling
Anti-p-mTOR	1:1000	2971	Cell Signaling
Anti-GAPDH	1:4000	2118	Cell Signaling
Anti-p-p53	1:1000	9284	Cell Signaling

Table S2. Primers for qPCR

**Name**	**Sequence**	**Reference**
PCNA-F	TTTGGTGCAGCTCACCCTG	(13)
PCNA-R	CGCGTTATCTTCGGCCCTTA	
BCL-2-F	CTGGTGGACAACATCGCCCT	
BCL-2-R	TCTTCAGAGACAGCCAGGAGAAAT	
BAX-F	CAAACTGGTGCTCAAGGCCC	
BAX-R	GGGCGTCCCAAAGTAGGAGA	
p53-F	GCCCAACAACACCAGCTCCT	(14)

p53-R	CCTGGGCATCCTTGAGTTCC	
GAPDH-F	CCTCAAGATCATCAGCAATGCC	self-designed
GAPDH-R	ACAGTCTTCTGGGTGGCAGT	

References

13. Amatori S, Persico G, Fanelli M. Real-time quantitative PCR array to study drug-induced changes of gene expression in tumor cell lines. J Cancer Metastasis Treat. 2017;3:90-9.

14. Phan RT, Dalla-Favera R. The BCL6 proto-oncogene suppresses p53 expression in germinal-centre B cells. Nature. 2004;432(7017):635-9.
